# Sleep Apnea in Arthritis Patients: A Multifactorial Analysis in the National Alzheimer’s Coordinating Center Cohort

**DOI:** 10.1002/alz.089341

**Published:** 2025-01-03

**Authors:** Bilal Irfan, Subhamoy Pal, Jonathan M Reader, Bruno Giordani

**Affiliations:** ^1^ Michigan Alzheimer’s Disease Research Center, Ann Arbor, MI USA; ^2^ University of Michigan Medical School, Ann Arbor, MI USA

## Abstract

**Background:**

Sleep apnea, a common sleep disorder, has been associated with various health conditions, including arthritis. This study investigates the relationship between sleep apnea and arthritis, examining how demographic and clinical characteristics impact this association. There are several interrelations between sleep apnea and arthritis, one of which may be attributed to systemic inflammation and oxidative stress pathways commonly activated in both conditions. There is an increasing prevalence of both conditions in aging populations, with potential implications for clinical practice and patient management.

**Method:**

The study utilized logistic regression analysis to assess the presence of sleep apnea in a sample of 46,268 participants from NACC ADRC first assessments with UDSv3, considering arthritis, age, race (white/non‐white), Hispanic origin, sex, education, and diagnosis (Alzheimer’s Disease [AD], Mild Cognitive Impairment [MCI], Cognitively Unimpaired). The analysis aimed to understand the likelihood of sleep apnea occurrence in relation to these factors.

**Result:**

The mean age of participants was 71.33 years (57% female). Arthritis was significantly associated with sleep apnea (aOR = 1.91, 95% CI: 1.75, 2.09, p < 0.001). Age showed a borderline non‐significant influence (aOR = 1.00, p = 0.086). Female sex was less likely to be associated with sleep apnea compared to males (aOR = 0.40, 95% CI: 0.37, 0.44, p < 0.001). Educational attainment showed a slight positive correlation with sleep apnea presence (aOR = 1.02, p = 0.018). In terms of cognitive diagnosis, MCI and AD were associated with higher odds of sleep apnea compared to normal cognition (MCI: aOR = 1.33, p < 0.001; AD: aOR = 1.13, p = 0.026). MCI was associated with higher odds of sleep apnea compared to AD (aOR = 1.18, p = 0.004). Race and Hispanic origin did not show significant associations with sleep apnea.

**Conclusion:**

The study highlights a significant association between arthritis and sleep apnea. Sex differences and educational level also play a role in the likelihood of developing sleep apnea among arthritis patients. These findings suggest a need for targeted screening and management strategies for patients with both sleep apnea and arthritis, particularly considering sex and cognitive status.

